# The Need of a National Dental Board of Registration

**Published:** 1918-06

**Authors:** M. Dewey


### THE NEED OF A NATIONAL DENTAL BOARD OF REGISTRATION

BY M. DEWEY, D.D.S.

For a number of years a few men in the dental profession have realized that the present plan of registration of dentists by individual state boards does not possess all of the advantages that are desired by the profession as a whole. Under the present conditions, the registration of dentists is controlled by the state board of each state, with the result that a man may be qualified to practice dentistry in one state, and by not fulfilling requirements of the board in another state, he is prevented from going to a different field to practice, although conditions seem more favorable for him to make the change.

We realize that according to the present law, each state can make whatever restrictions it desires in regard to the registration of men engaged in the practice of dentistry. This is based upon the old question of "state rights," but we believe that such important bodies as the medical and dental professions should not be subservient to the individual "state rights" or state laws, but should be governed by a national dental board of registration which would have the same plan, laws, and regulations governing the entire medical and dental professions of the United States. We are aware of the fact that under certain conditions state boards have made concessions under the term of "reciprocity," which have some advantages, and which also have a great many disadvantages. There are certain states in the Union that are very much opposed to dentists from any other locality coming to those states to practice. In such states we find the dental boards have refused to make reciprocal arrangements with other boards. In fact, the state dental board has taken upon itself the function of protecting the men registered in the state against too large a number of men being engaged in the practice of dentistry. In some instances state boards have attempted to build up lines of restriction which would prevent anyone from being permitted to practice in the state unless he is a native son or graduate from a certain school. This may be a satisfactory arrangement so far as the men already registered in the state are concerned, but it is a very small, selfish, and unsatisfactory plan in relation to the dental profession of the United States as a whole.

Again we see the need of a national dental board of registration as a result of conditions which have been produced by the war. We find men in the dental profession, from all localities who have given their service to the country, with the result that there are a great many dental offices vacant, and in some communities the number of dentists has been so decreased that there are not enough left to take care of the public. We find some instances where a man who joined the army has been desirous of having some one in his office to take care of his practice while he is away, and the man he desired to bring into the office is not registered in that state. Consequently, his dental practice has been left without anyone to take care of it, simply because the dental laws of the United States are so arranged that a man who is registered in one state is not permitted to occupy an office in another, even though the man who owns the original office is rendering a service to the country by giving his time to the army or navy.

We have known of several instances in which men engaged in the practice of orthodontia have given up their practice to join the army, and have attempted to get orthodontists from other states to take care of their practices while they were gone; but, of course, were prevented from doing so by the state boards. These men who have given their services to the country may be away from their offices for a number of years; and as a result of having no one in the office, their practice will be in bad shape when they return.

In other instances men in the army and navy may desire to change their location when they return to civilian practice due to the fact that it will be practically as easy to build up a practice in one locality as it will be to build up a practice by returning to their old location. We believe that out of fairness to those men who have given their service to the country, some provision should be made whereby they can locate in any community they desire, even though it be in a different state than the one in which they formerly practiced. We realize this matter is going to be very difficult to arrange with the present system of individual state boards. With a national dental board the matter could be very easily arranged. We have never been able to see why a man who is qualified to practice dentistry in one state is not qualified to practice in another. The only possible reason we can find for the existence of a number of state boards is the old question of state rights, plus the fact that each individual state board has a very exalted opinion of itself and believes it occupies a very important position in the dental profession, along with the political preference which the board gets by its existence.

We believe a great step will be made in the advance of the dental profession as a national institution when state boards are eliminated and the registration of dentists is placed under one national board and probably controlled by the Surgeon-General's office. We have given this matter much thought in times past, and can come to no other conclusion except that a national board of registration will be a decided advantage over the present plan of individual registration in each state by local state boards. We hope that we may see the time when such a board will be a certainty, and in existence, rather than a need and a desire.—*International Journal of Orthodontia.*

